# Global warming not so harmful for all plants - response of holomycotrophic orchid species for the future climate change

**DOI:** 10.1038/s41598-017-13088-7

**Published:** 2017-10-05

**Authors:** Marta Kolanowska, Marta Kras, Monika Lipińska, Katarzyna Mystkowska, Dariusz L. Szlachetko, Aleksandra M. Naczk

**Affiliations:** 1 Department of Plant Taxonomy and Nature Conservation, Faculty of Biology, University of Gdansk, Wita Stwosza 59, 80308 Gdańsk, Poland; 2 Department of Biodiversity Research, Global Change Research Institute AS CR, Bělidla 4a, 60300 Brno, Czech Republic; 3 Department of Molecular Evolution, Faculty of Biology, University of Gdańsk, Wita Stwosza 59, 80308 Gdańsk, Poland

## Abstract

Current and expected changes in global climate are major threat for biological diversity affecting individuals, communities and ecosystems. However, there is no general trend in the plants response to the climate change. The aim of present study was to evaluate impact of the future climate changes on the distribution of holomycotrophic orchid species using ecological niche modeling approach. Three different scenarios of future climate changes were tested to obtain the most comprehensive insight in the possible habitat loss of 16 holomycotrophic orchids. The extinction of *Cephalanthera austiniae* was predicted in all analyses. The coverage of suitable niches of *Pogoniopsis schenckii* will decrease to 1–30% of its current extent. The reduction of at least 50% of climatic niche of *Erythrorchis cassythoides* and *Limodorum abortivum* will be observed. In turn, the coverage of suitable niches of *Hexalectris spicata*, *Uleiorchis ulaei* and *Wullschlaegelia calcarata* may be even 16–74 times larger than in the present time. The conducted niche modeling and analysis of the similarity of their climatic tolerance showed instead that the future modification of the coverage of their suitable niches will not be unified and the future climate changes may be not so harmful for holomycotrophic orchids as expected.

## Introduction

Current and expected changes in global climate are major threat for biological diversity affecting individuals, communities, ecosystems and ecoregions^1,2^. The recent studies confirmed that plant distribution is determined by climatic factors and while the temperature-related stress controls the upper-latitudinal and upper-altitudinal range limits of a large proportion of many plant species, other variables (e.g. water deficiency stress) may be important at the lower range limits^3,4^. However, there is no general trend in the plants response to the climate change^5^. The Intergovernmental Panel on Climate Change (IPCC) predicted that increase in global mean temperature of 1–3 °C above 1990 levels will give a beneficial impacts in some regions and harmful ones in others. Global climate change has already had observable negative effects on the nature, e.g. shrunk of the glaciers, more frequent fires, longer periods of water deficiency in some regions and an increase in the number, duration and intensity of tropical storms. However, also positive impact on environment are expected be witnessed. Higher CO_2_ level is likely to be beneficial to many plants, resulting in an acceleration of biomass production, increased precipitation may also benefit some species^6^.

Orchidaceae is among the largest flowering plant families which is currently facing exceptional risks of extinction^7,8^ and despite conservation efforts, numerous orchid populations continue to decline^9–13^. Orchids are susceptible to habitat fragmentation due to their unique reproductive strategies, specific interactions with symbionts, and often specific habitat requirements. Effective conservation actions require the identification of areas characterized by suitable habitat conditions in order to facilitate prioritization and determination of zones suitable for creation of reserves^11,14^. For poorly known species, often with incomplete distribution records, species distribution models are invaluable tools facilitating the selection of priority conservation areas^15–17^. Unfortunately, little research concerned the future changes in the suitable climatic niche coverage of orchid species. Preliminary studies showed that numerous representatives of *Dactylorhiza* Neck. *ex* Nevski^18^, *Epipactis helleborine* (L.) Crantz^19^ and invasive *Arundina graminifolia* (D. Don) Hochr.^20^ will face reduction of their preferred climatic niches extension.

While most representatives of Orchidaceae are epiphytes, the majority of threatened species are terrestrial plants, often characterized by specialized habitat requirements^21,22^. A distinctive group within terrestrial orchids are holomycotrophic species which represent only 33 genera^23^ of over 700 genera which are currently recognized^24^. These plants are found throughout the globe from the Arctic regions of North America, Greenland, Iceland, Scandinavia and Siberia to Southern Africa, Australia and New Zealand. Their greatest diversity is observed in Asia and west Pacific region (170 species representing 24 genera). Holomycotrophic orchids were not reported from the southern regions of South America, the Atlantic islands (including Macaronesia) and the Subantarctic region^23^.

The aim of present study was to evaluate impact of the future climate changes on the distribution of holomycotrophic orchids using ecological niche modeling approach. The estimated changes in the suitable niches coverage of studied species were compared with their climatic preferences similarity to reveal any common aspect of their climatic tolerance that would be favorable in the future.

## Results

### Ecological niche models evaluation

The calculated AUC values were high for all created models (Supplementary Table S1). Most of them are consistent with the actually known geographical range of the studied species (Supplementary Figs S1–S6). Discrepancies are observed in model of *C. wisteriana* which show low suitability of climatic niche in areas where the species is known to occur. Models of *P. schenckii*, *U. ulaei*, *W. calcarata*, *A. montana*, *G. lindleyana*, and *A. macranthus* showed additional areas of high suitability in places where no records of these species were reported so far, however it is commonly accepted that realized niche is smaller than the climatic niche.

### Factors limiting occurrence

The most limiting distribution factor of studied orchids was precipitation of the driest month (bio14). This variable was crucial for occurrence of six studied species: *H. spicata, W. calcarata, D. hamiltonianum, E. cassythoides, N. nidus-avis*, and *E. aphyllum*. The precipitation of the coldest quarter (bio19) significantly contributed in the models for *C. austiniae, U. ulaei*, and *G. sesamoides*. Occurrence of two species, *C. wisteriana* and *P. schenckii*, depends mostly on the maximum temperature of the warmest month (bio5). The annual precipitation (bio12) was the most important limiting factor for *A. montana*, the precipitation of the warmest quarter (bio18) for *G. lindleyana*, and the mean diurnal range (bio2) for *A. macranthus*. The complete list of the variables contributing to the models of studied species is given in Supplementary Table S2.

### Variation of climatic preferences

**Figure 1:**
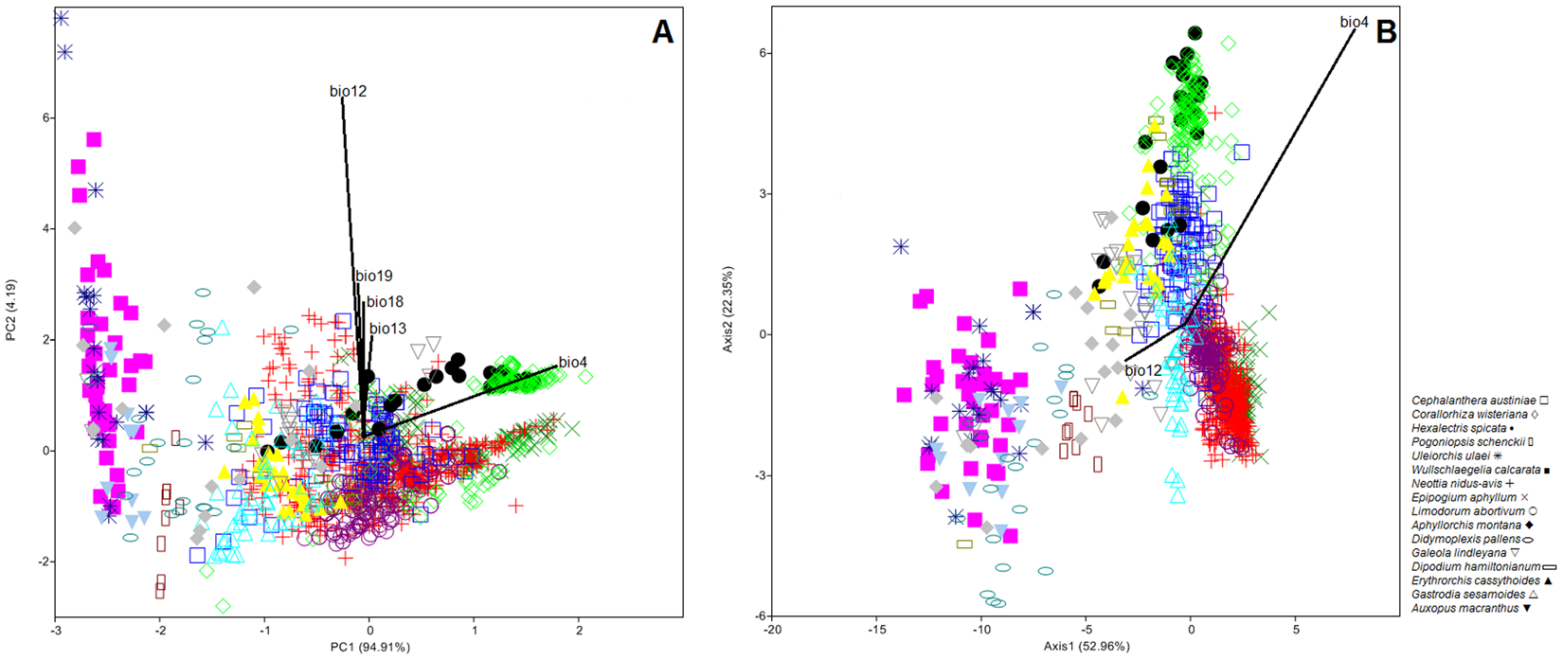
Principal components analysis (PCA) of the studied holomycotrphic orchids based on the bioclimatic factors from individuals. Variables with the greatest contributions are presented as vectors (**A**). Canonical variates analysis (CVA) of the studied holomycotrophic species along environmental gradient. The relative contributions of bioclimatic factors are shown as vectors. Variables with low discriminating impact are omitted (**B**). Diagrams were prepared in PAST 2.14 (https://folk.uio.no/ohammer/past/).

The bioclimatic variation of the studied orchids visible on the PCA analysis diagrams was large. The specimens have created almost linear pattern of grouping along environmental gradient (Fig. 1A). The first axis distinguished *G. lindleyana*, *E. aphyllum*, *H. spicata* (above the axis) from *P. schenckii* and *L. abortivum* (below the axis). Furthermore, the second axis separated *E. aphyllum* and *C. wisteriana* from *P. schenckii*, *U. ulaei*, *W. calcarata*, *A. montana*, *D. pallens*, *D. hamiltonianum*, *E. cassythoides*, *G. sesamoides*, and *A. macranthus* on the left side of the diagram. Variables with the greatest contributions enhanced in the overall analysis were: the temperature seasonality (bio4); the annual precipitation (bio12); the precipitation of the wettest month (bio13); the precipitation of the warmest quarter (bio18); the precipitation of the coldest quarter (bio19). In turn, the canonical variate analysis (CVA) showed a highly statistically significant differentiating value for the holomycotrophic species with respect to the bioclimatic factors (Wilks λ = 0.001; F_(180;12807)_ = 75.381; *P* < 0.0001). The canonical variate axes (52.96 and 22.35% of explained variance, respectively) have distinguished individual species from each other along environmental gradient obtained on the basis of the bioclimatic factors (Fig. 1B).

The major share in the discrimination of the studied species had the temperature seasonality (bio4) and the annual precipitation (bio12) which discriminated *C. austiniae* and *C. wisteriana*. Isothermality (bio3 = −1.197), the precipitation of the coldest quarter (bio19 = 0.704), as well as, the annual mean temperature (bio1 = 3.137), the mean diurnal range of the temperature (bio2 = 1.971) and the maximum temperature of the warmest month (bio5 = −2.983) had also the significant meaning in the performed discrimination of the studied orchids. The bioclimatic differences between the pairs of species were evaluated by permutation tests of the climatic factors. The interaction term in the MANOVA (Pillai’s trace = 3.843; F_(180;16380)_ = 42.88; *P* < 0.0001) was significant. The tests demonstrated significant differences of the climatic factors preferences in all species pairs with permutation of *P* < 0.0001, except for the pair of species, *D. hamiltonianum* and *E. cassythoides*.

**Figure 2:**
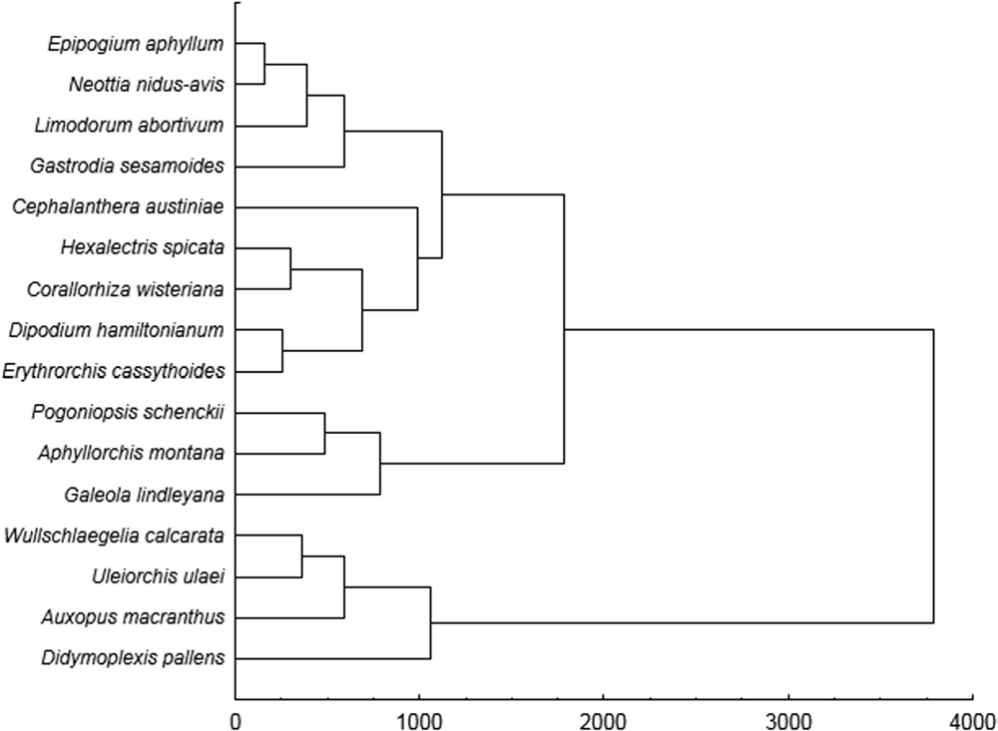
The cluster analysis UPGMA of the studied holomycotrophic species in terms bioclimatic differences. Diagram was prepared in PAST 2.14 (https://folk.uio.no/ohammer/past/).

Two clades were recognized in the UPGMA dendrogram (Fig. 2) illustrating the similarity between analysed orchids in the preferred niches. Both included species from different geographical regions.

### Future changes in the suitable niche coverage

The changes in the coverage of the most suitable climatic niches of studied holomycotrophic orchids (Figs 3–20) are presented in Supplementary Table S3. The niche extension of nine species will increase in A1b (Figs 3–8) and B2a (Figs 15–20) climate change scenarios. Due to the climate modification described in these scenarios seven species will experience the suitable climatic niche loss. In A2a (Figs 9–14) scenario the coverage of niche of eight species will decrease and for other eight orchids (*H. spicata, U. ulaei, W. calcarata, D. hamiltonianum, D. pallens, N. nidus-avis, E. aphyllum, A. macranthus*) the changes will be favorable.

**Figure 3:**
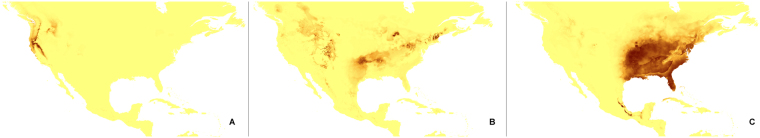
Predicted distribution of suitable climatic niches of *Cephalanthera austiniae* (**A**), *Corallorhiza wisteriana* (**B**), *Hexalectris spicata* (**C**) in 2080 according to A1b scenario. Maps were generated in ArcGis 9.3^76^ (http://www.esri.com/).

**Figure 4:**
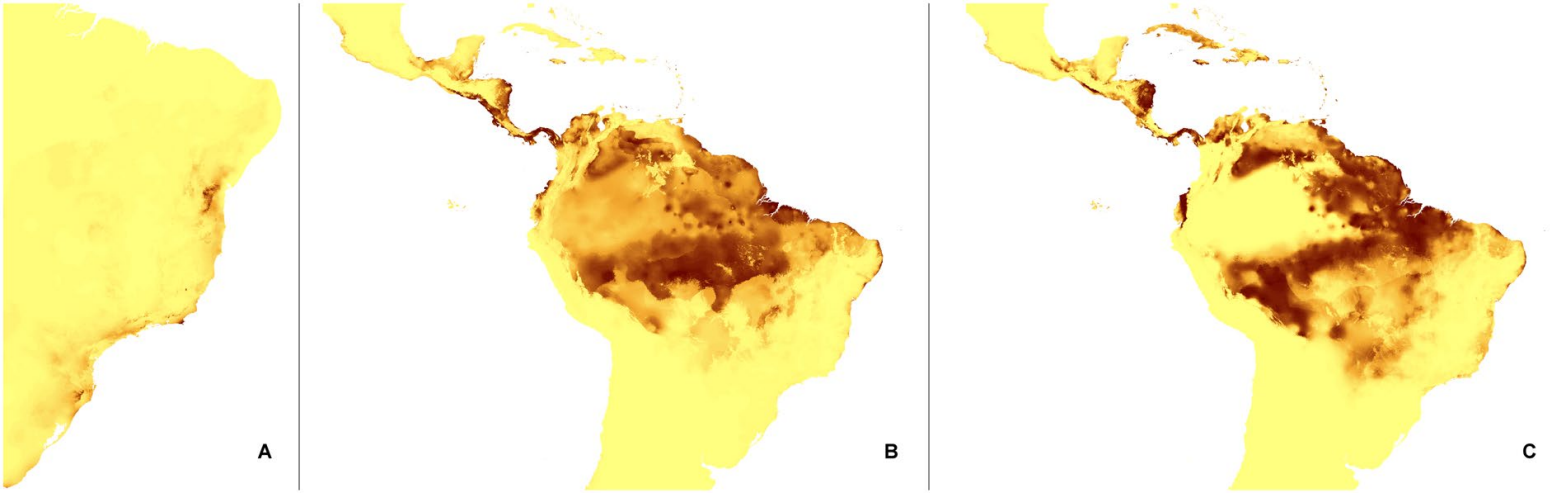
Predicted distribution of suitable climatic niches of *Pogoniopsis schenckii* (**A**), *Uleiorchis ulaei* (**B**), *Wullschlaegelia calcarata* (**C**) in 2080 according to A1b scenario. Maps were generated in ArcGis 9.3^76^ (http://www.esri.com/).

**Figure 5:**
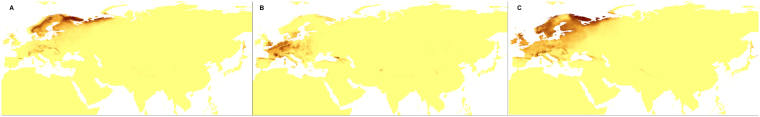
Predicted distribution of suitable climatic niches of *Neottia nidus-avis* (**A**), *Epipogium aphyllum* (**B**), *Limodorum abortivum* (**C**) in 2080 according to A1b scenario. Maps were generated in ArcGis 9.3^76^ (http://www.esri.com/).

**Figure 6:**
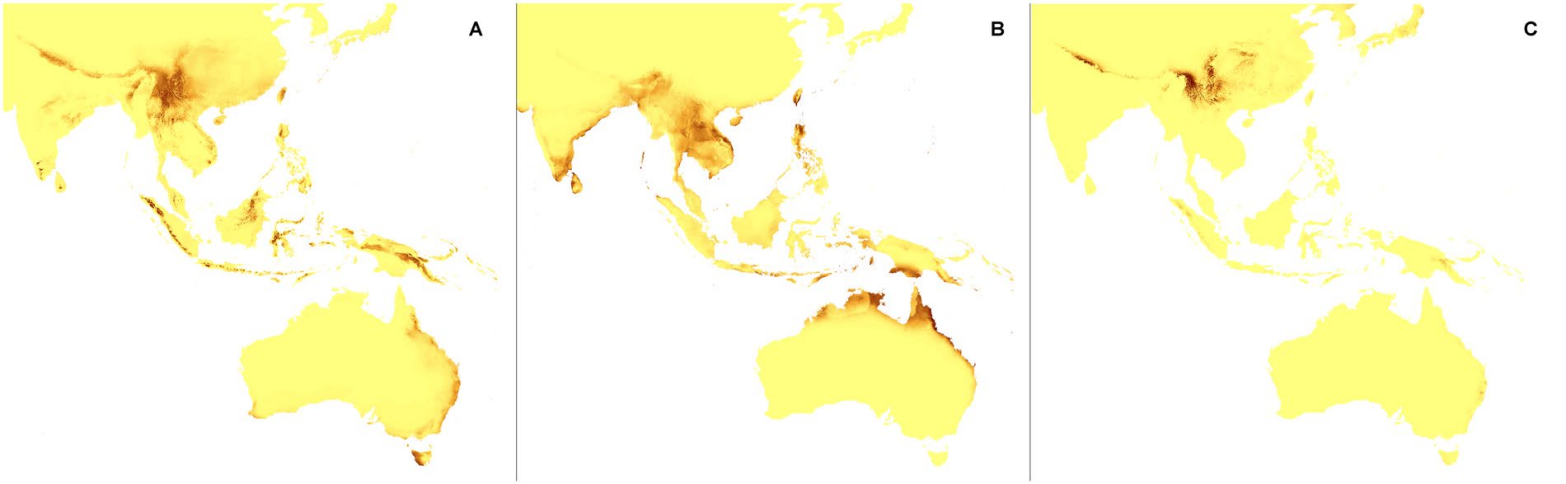
Predicted distribution of suitable climatic niches of *Aphyllorchis montana* (**A**), *Didymoplexis pallens* (**B**), *Galeola lindleyana* (**C**) in 2080 according to A1b scenario. Maps were generated in ArcGis 9.3^76^ (http://www.esri.com/).

**Figure 7:**
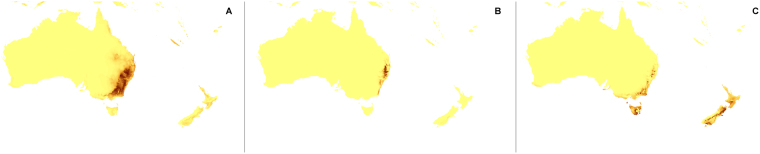
Predicted distribution of suitable climatic niches of *Dipodium hamiltonianum* (**A**), *Erythrorchis cassythoides* (**B**), *Gastrodia sesamoides* (**C**) in 2080 according to A1b scenario. Maps were generated in ArcGis 9.3^76^ (http://www.esri.com/).

**Figure 8:**
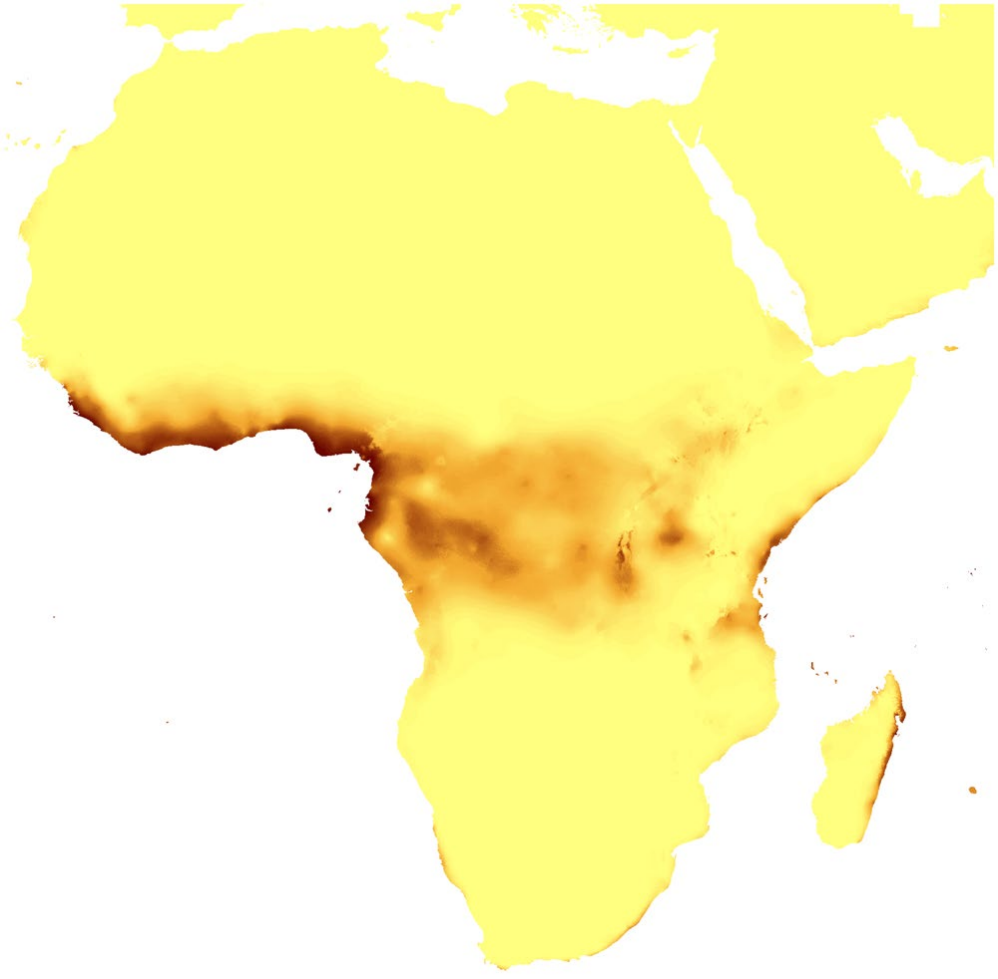
Predicted distribution of suitable climatic niches of *Auxopus macranthus* in 2080 according to A1b scenario. Maps were generated in ArcGis 9.3^76^ (http://www.esri.com/).

**Figure 9:**
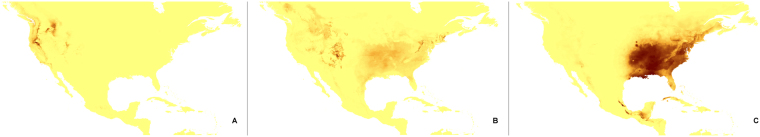
Predicted distribution of suitable climatic niches of *Cephalanthera austiniae* (**A**), *Corallorhiza wisteriana* (**B**), *Hexalectris spicata* (**C**) in 2080 according to A2a scenario. Maps were generated in ArcGis 9.3^76^ (http://www.esri.com/).

**Figure 10:**
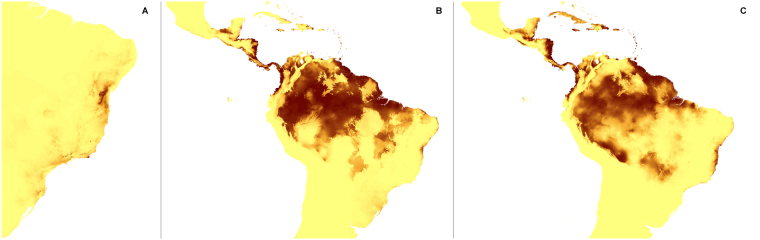
Predicted distribution of suitable climatic niches of *Pogoniopsis schenckii* (**A**), *Uleiorchis ulaei* (**B**), *Wullschlaegelia calcarata* (**C**) in 2080 according to A2a scenario. Maps were generated in ArcGis 9.3^76^ (http://www.esri.com/).

**Figure 11:**
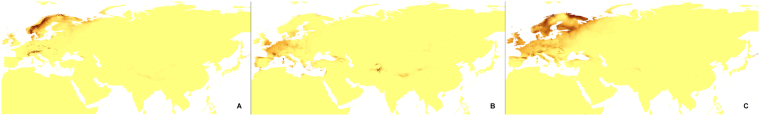
Predicted distribution of suitable climatic niches of *Neottia nidus-avis* (**A**), *Epipogium aphyllum* (**B**), *Limodorum abortivum* (**C**) in 2080 according to A2a scenario. Maps were generated in ArcGis 9.3^76^ (http://www.esri.com/).

**Figure 12:**
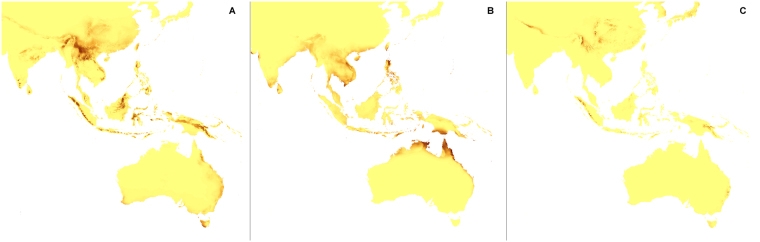
Predicted distribution of suitable climatic niches of *Aphyllorchis montana* (**A**), *Didymoplexis pallens* (**B**), *Galeola lindleyana* (**C**) in 2080 according to A2a scenario. Maps were generated in ArcGis 9.3^76^ (http://www.esri.com/).

**Figure 13:**
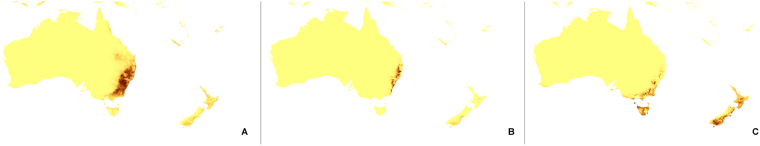
Predicted distribution of suitable climatic niches of *Dipodium hamiltonianum* (**A**), *Erythrorchis cassythoides* (**B**), *Gastrodia sesamoides* (**C**) in 2080 according to A2a scenario. Maps were generated in ArcGis 9.3^76^ (http://www.esri.com/).

**Figure 14:**
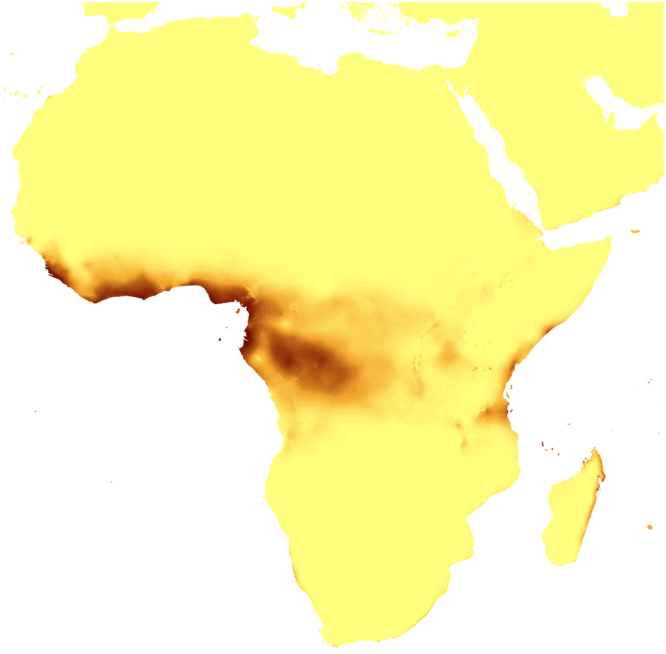
Predicted distribution of suitable climatic niches of *Auxopus macranthus* in 2080 according to A2a scenario. Maps were generated in ArcGis 9.3^76^ (http://www.esri.com/).

**Figure 15:**
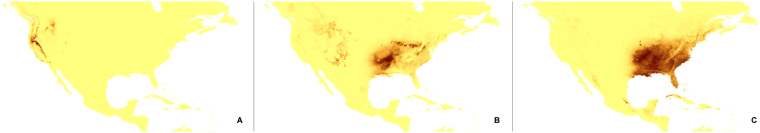
Predicted distribution of suitable climatic niches of *Cephalanthera austiniae* (**A**), *Corallorhiza wisteriana* (**B**), *Hexalectris spicata* (**C**) in 2080 according to B2a scenario. Maps were generated in ArcGis 9.3^76^ (http://www.esri.com/).

**Figure 16:**
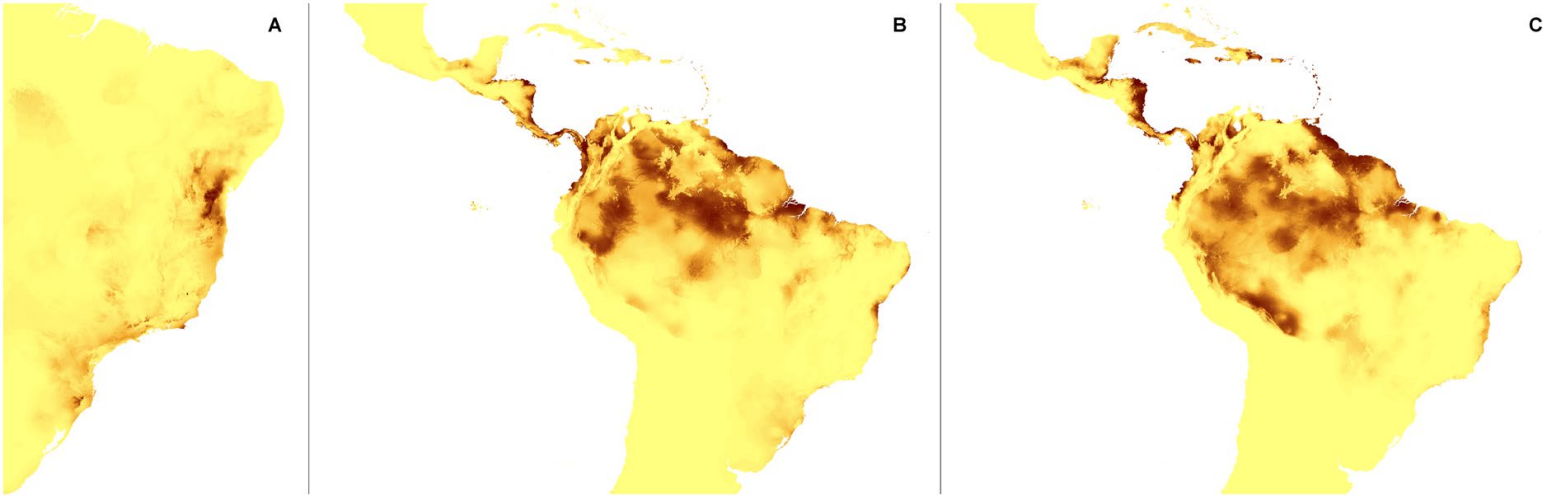
Predicted distribution of suitable climatic niches of *Pogoniopsis schenckii* (**A**), *Uleiorchis ulaei* (**B**), *Wullschlaegelia calcarata* (**C**) in 2080 according to B2a scenario. Maps were generated in ArcGis 9.3^76^ (http://www.esri.com/).

**Figure 17:**
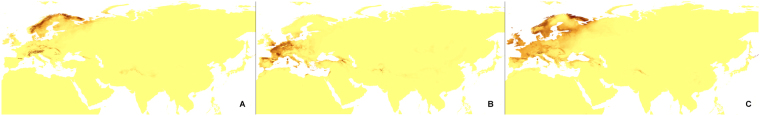
Predicted distribution of suitable climatic niches of *Neottia nidus-avis* (**A**), *Epipogium aphyllum* (**B**), *Limodorum abortivum* (**C**) in 2080 according to B2a scenario. Maps were generated in ArcGis 9.3^76^ (http://www.esri.com/).

**Figure 18:**
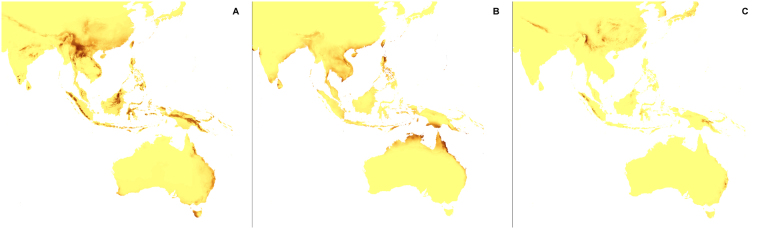
Predicted distribution of suitable climatic niches of *Aphyllorchis montana* (**A**), *Didymoplexis pallens* (**B**), *Galeola lindleyana* (**C**) in 2080 according to B2a scenario. Maps were generated in ArcGis 9.3^76^ (http://www.esri.com/).

**Figure 19:**
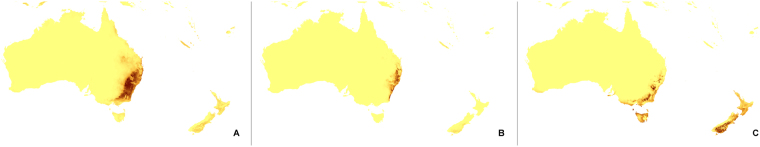
Predicted distribution of suitable climatic niches of *Dipodium hamiltonianum* (**A**), *Erythrorchis cassythoides* (**B**), *Gastrodia sesamoides* (**C**) in 2080 according to B2a scenario. Maps were generated in ArcGis 9.3^76^ (http://www.esri.com/).

**Figure 20:**
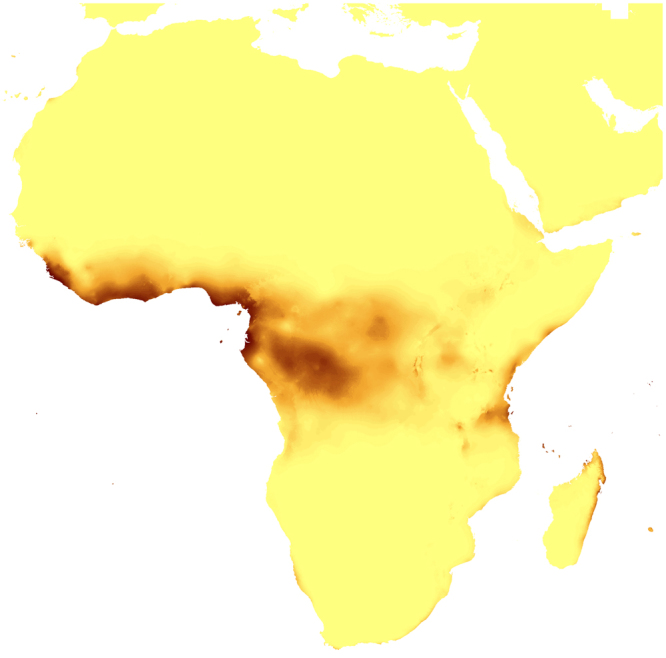
Predicted distribution of suitable climatic niches of *Auxopus macranthus* in 2080 according to B2a scenario. Maps were generated in ArcGis 9.3^76^ (http://www.esri.com/).

The suitable climatic niches of seven species, *H. spicata, U. ulaei, W. calcarata, D. hamiltonianum, D. pallens, N. nidus-avis*, and *A. macranthus* will not be negatively affected by climate changes in any scenario. Five, *C. austiniae, P. schenckii, E. cassythoides, A. montana*, and *L. abortivum* will lose suitable niches in all scenarios. The negative effect on climatic niche extension of *C. wisteriana* and *G. sesamoides* will be observed in A1b and A2a scenarios. Climatic niche loss will be observed in case of *G. lindleyana* in A2a and B2a scenarios while *E. aphyllum* will lose niches exclusively in B2a scenario.

The negative effect of climate change will be remarkable for *C. austiniae* and *P. schenckii*. The extinction of *C. austiniae* was predicted in all analyses. The coverage of suitable niche of *P. schenckii* will decrease to 1-30% of its current extent. The reduction of at least 50% of climatic niche coverage of *E. cassythoides* and *L. abortivum* will be observed. On the other hand, the coverage of suitable niches of *H. spicata, U. ulaei* and *W. calcarata* may be even 16–74 times larger than in the present time.

## Discussion

Predicting the response of biodiversity to climate change became an important aspect of nature conservation^2,25–28^. The computed models are crucial for warning decision makers to potential future risks and support the establishment of active strategies to reduce negative impacts of climate change on organisms^26,29,30^. Climate change scenarios are the base for predictive analysis and these depend on a various socio-economic storylines for greenhouse gas emissions^26^ and on a broad range of General Circulation Models used to calculate climate change for given trajectories of greenhouse gas emissions^30^. For this reason the projections of habitat loss can gave a contrasting results depending on the choice of combinations of emissions scenarios and climate models^31^. Bellgard & Williams^32^ partitioned the global climate change into four putative time episodes. According to their proposal the long-term impacts (over 21–50+ years) are related with increased temperatures and CO_2_ that will destabilize global rainfall patterns, soil properties and plant ecosystem resilience. Because of dependence on their host for C-supply, orchid mycorrhizas and all heterotrophic mycorrhizal groups will be immediately impacted through loss of habitat and plant-hosts.

In our study three different scenarios of future climate changes were tested to obtain the most comprehensive insight in the possible climatic niche loss of 16 species of holomycotrophic orchids. No general trend in the response of holomycotrophic orchid for climate changes was identified. We did not recognize any correlation between climatic factors currently limiting the distribution of the studied orchids with their response to climate changes. The first of two clades recognized in the UPGMA dendrogram included 12 species from different geographical regions (Europe, Asia, Australia, South and North America) which suitable niche coverage will be variously modified in the future. The second clade included species which potential niche coverage will increase as a result of climate change - two South American species (*W. calcarata* and *U. ulaei*), African *A. macranthus* and Asian *D. pallens*. This analysis indicate that species similar in general climatic preferences will respond to the global warming in different way.

Obviously, the actual future range of studied orchids may be slightly different than presented in our models. The availability of mycorrhizal fungi and presence of pollinators may restrict the distribution of these species while the niche shift may allow them to occupy new habitats. The current state of knowledge on the specify of holomycotrophic orchid symbionts and their dependence on particular pollen carriers is unfortunately too incomplete to include these elements in the analyses. Moreover, such complex ecological process are not possible to evaluate using currently available statistic approaches. However, we do believe that climate is the key factor limiting occurrence of most organisms by determining characteristics and distributions of natural and managed systems. Hereby modeling of distribution of climatic niches is the first step to evaluate the response of particular species to the possible climate changes.

It is not clear how and whether the function of mycorrhizal fungi in orchid germination and growth will be maintained with rising temperature, erratic rainfall and reduced moisture. The research conducted on photosynthetic, terrestrial orchids from Europe - *Anacamptis morio* (L.) R.M. Bateman, Pridgeon & M.W. Chase and *Dactylorhiza fuchsii* (Druce) Soó indicated that orchid rarity and persistence are not necessarily related to fungal diversity and that other factors may be more important in determining orchid survival chances^33^. Furthermore, the modifications of the soil structure which is especially important for terrestrial plants are extremely difficult to evaluate due to multiple factors which can influence the ground properties. Studies on European plants growing in understory layer indicated that numerous species are able to shift their realized niches, also for soil nutrients and pH^34^. The climate change (i.e. CO_2_ enrichment, arise temperature, altered precipitation, increased N-deposition) impact the soil-rhizosphere, plant and fungal physiology and/or ecosystem(s) directly and indirectly. Direct effects include changes in resource availability and change in distribution of mycorrhizas. Indirect effects include changes in below ground allocation of C to roots and changes in plant species distribution^32^.

As mentioned before the pollinators availability can limit the long term viability of orchid populations, but this factor could not be included in our analyses due to the lack of sufficient data on pollinator specificity of studied plants. We know at least five species of insects that can pollinate *E. aphyllum* in Poland and the Czech Republic^35^, but the complete set of species which are able to transfer pollinia of this orchid remains unknown, in turn, the recent study proved that the turnover in the composition of insect communities may occur in the future^36^. On the other hand, almost no data on pollinators of tropical Orchidaceae has been published. The estimation of the impact of future climate changes on the pollinators distribution in not the only problem because, as indicated by Robbirt *et al*.^37^ climate changes can also led to asynchrony in flowering of orchids and insect flight periods. The future modification in insects phenology are related with early adult emergence^38–40^, earlier larval emergence^41^, and earlier migration^40,42^. Additionally, the latitudinal^43,44^ and altitudinal^45,46^ shifts are predicted as a result of climate change. Also, the global expansion of tropical species into temperate areas is possible^47,48^.

Orchids as a group show evolutionary flexibility whereby diversification in the family is often related to habitat complexity and fragmentation. Throughout their history, Orchidaceae have been able to cope with face of climatic change caused by shifting continents, mountain uplifting, fluctuating sea levels and temperatures^49^. All these phenomena occur today but the rate of change seems to be occurring faster than the detectable past with available methods^50^. Some species experiences loss of habitat, population reduction and cannot keep pace with the climate change^51,52^. Liu *et al*.^53^ estimated that populations of at least 15% of the orchid species in a diverse region of southwestern China will be threatened with extinction over the next two centuries given projected climate changes. Also, Nadkarni & Solano^54^ and Olaya-Arenas *et al*.^55^ indicated drying trend in some cloud forests, including Costa Rica, which might affect those species such as the hundreds of Neotropical *Lepanthes* representatives that depend on cool and wet conditions.

Our studies indicated that the future climate changes may be not so harmful for all holomycotrophic orchids as expected^8^. Out of the 16 studied species, the potential suitable niches was not negatively affected in all the models. In our opinion the most significant threat for the plants is the direct human activity that results in habitat destruction and fragmentation or modification of the ecosystem net. The other conclusion of our study is that generalization of the effect of global warming is misleading. Not all organisms, even related, occurring in the same geographical region and preferring similar climatic conditions will face the same modifications of their suitable niches. The object of our study were holomycotrophic orchids that in theory should respond in a similar way for a global warming. The conducted niche modeling and analysis of the similarity of their climatic tolerance showed instead that the future modification of the coverage of their suitable niches will not be unified.

## Methods

### Species selection and list of localities

A total of 16 species representing 16 different genera were included in the study. We selected representatives of all three holomycotrophic genera from North America, i.e. *Cephalanthera austiniae* (A. Gray) A. Heller, *Corallorhiza wisteriana* Conrad, and *Hexalectris spicata* (Walter) Barnhart; three of four South American genera - *Pogoniopsis schenckii* Cogn., *Uleiorchis ulaei* (Cogn.) Handro, and *Wullschlaegelia calcarata* Benth., and members of three European genera - *Neottia nidus-avis* (L.) Rich., *Epipogium aphyllum* Sw., and *Limodorum abortivum* (L.) Sw. From numerous holomycotrophic species occurring in Asia we choose *Aphyllorchis montana* Rchb.f., *Didymoplexis pallens* Griff., and *Galeola lindleyana* (Hook.f. & J.W. Thomson) Rchb.f. Australian orchids are represented in this study by *Dipodium hamiltonianum* F.M. Bailey, *Erythrorchis cassythoides* (A.M. Cunn.) Garay, and *Gastrodia sesamoides* R. Br. From 18 African species we were able to gather sufficient number of localities only for *Auxopus macranthus* Summerh.

Over 1500 localities of studied orchids were compiled based on available herbarium material, literature data and electronic databases (Supplementary Dataset S1). Only those localities which could be precisely localized on the map were used. From the database the duplicate records were removed. To reduce the sampling bias we removed also closely lying localities. The final database (Supplementary Dataset S2) included a total of 1382 records: 93 of *C. austiniae*, 160 of *C. wisteriana*, 24 of *H. spicata*, 10 of *P. schenckii*, 21 of *U. ulaei*, 37 of *W. calcarata*, 17 of *D. hamiltonianum*, 29 of *E. cassythoides*, 55 of *G. sesamoides*, 17 of *A. montana*, 22 of *D. pallens*, 21 of *G. lindleyana*, 481 of *N. nidus-avis*, 190 of *E. aphyllum*, 191 of *L. abortivum* and 14 of *A. macranthus*. This is more than the minimum number of records required to obtain reliable predictions in MaxEnt application^56,57^.

### Ecological niche modelling

While a broad range of algorithms^58,59^ and platforms (i.e. BIOMOD^60^, ModEco^61^, openModeller^62^) can be used to produce species distribution models, in this study the maximum entropy method implemented in MaxEnt v. 3.3.2^63–65^ based on the species presence-only observations was used. This application has been proved to provide the most robust response across the number of environmental variables tested^66^ and it has been shown to work better with small number of samples than other approaches^67^. From 19 climatic variables (“bioclims”; Supplementary Table S4) in 2.5 arc minutes (±21.62 km^2^ at the equator) developed by Hijmans *et al*.^68^ and provided by WorldClim (v. 1.4 release 3; www.worldclim.org) we removed seven variables due to their significant correlation. For the same reason we did not use altitude as input data. The variables included in the analysis are listed in Supplementary Table S4.

To improve model performance target-group background was selected for each continent^69^. Additionally for *P. schenckii* we reduced background to avoid significant over-estimation of the model (Supplementary Table S5).

In all analyses the maximum iterations was set to 10000 and convergence threshold to 0.00001. The “random seed” option was applied, this provides random test partition and background subset for each run was applied. A bootstrap procedure with 1000 replicates was applied and the output was set to logistic. All operations on GIS data were carried out on ArcGis v. 9.3 (ESRI).

To estimate the impact of hypothetical climate changes on the coverage of suitable niches of studied plants we used the same climatic variables as considered in the present models. The layers based on the coupled global climate models were used (http://ccafs-climate.org). Three various emission scenarios for 2080 were analysed: A1b (CCCMA-CGCM3 SRES simulation), A2a (CCCMA-CGCM2 SRES) and B2a (CCCMA-CGCM2 SRES). This approach was used in numerous recent studies that focused on the climate change impact on the distribution of various organisms^70–72^. While we are aware that recently several new models predicting future climate change have been published^73^, we used aforementioned datasets to compare our results with previously published studies focused on changes in coverage of climatic niche of Orchidaceae^18^.

### Multivariate analysis

Principal components analysis (PCA) was performed to explain the general variation pattern among the studied holomycotrophic species, based on 12 bioclimatic factors used in ENM analysis (Table S4) and altitudinal data. To determine the bioclimatic factors which differentiate the studied orchids the most, the canonical variate analysis (CVA) was applied, in order to reduce the data set by selecting only the factors that showed strongest discrimination. The significance of bioclimatic differences between the studied species was evaluated using Wilk’s λ and Goodall’s F-ratio with 1000 permutations. In turn, the Mahalanobis distance matrix was prepared to classification of examined species and was used in the cluster analysis UPGMA (unweighted pair-group average method)^74^. Statistical computations were performed with the program PAST v. 2.14^75^.

### Compliance with Ethical Standards

The authors declare that there are no conflicts of interest. This article does not contain any studies with human participants or animals performed by any of the authors.

## Electronic supplementary material

Supplementary information

Supplementary Dataset S1

Supplementary Dataset S2
