# Supplementary material for: Global warming not so harmful for all plants - response of holomycotrophic orchid species for the future climate change
Source: Sci Rep. 2017 Oct 5;7:12704. doi: 10.1038/s41598-017-13088-7 (PMC5629220; doi:10.1038/s41598-017-13088-7)
Supplement: Supplementary file 1 — Supplementary information [file 41598_2017_13088_MOESM1_ESM.pdf]

Global warming not so harmful for all plants - response of holomycotrophic orchid species for the future climate change

Marta Kolanowska[1,2], Marta Kras[1], Monika Lipińska[1], Katarzyna Mystkowska[1], Dariusz L. Szlachetko[1] & Aleksandra M. Naczk[3*]

[1]Department of Plant Taxonomy and Nature Conservation, Faculty of Biology, University of Gdansk, Wita Stwosza 59, 80308 Gdańsk, Poland; [2]Department of Biodiversity Research, Global Change Research Institute AS CR, Bělidla 4a, 60300 Brno, Czech Republic; and [3]Department of Molecular Evolution, Faculty of Biology, University of Gdańsk, Wita Stwosza 59, 80308 Gdańsk, Poland

[*]Correspondence and requests for materials should be addressed to A.M.N. (email: aleksandra.naczk@biol.ug.edu.pl, +48 58 523 60 49)

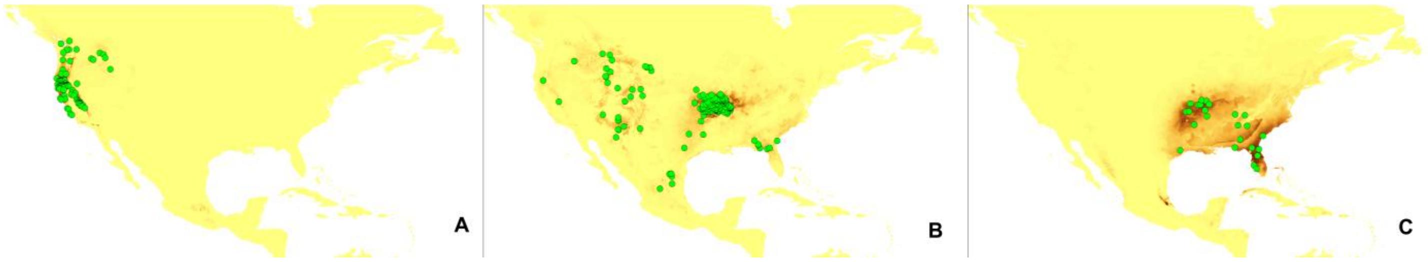

Supplementary Figure S1. Current distribution of suitable climatic niches of North American orchids included in the study: *Cephalanthera austiniae* (**A**), *Corallorhiza wisteriana* (**B**), *Hexalectris spicata* (**C**). Maps were generated in ArcGis 9.3[76] (http://www.esri.com/).

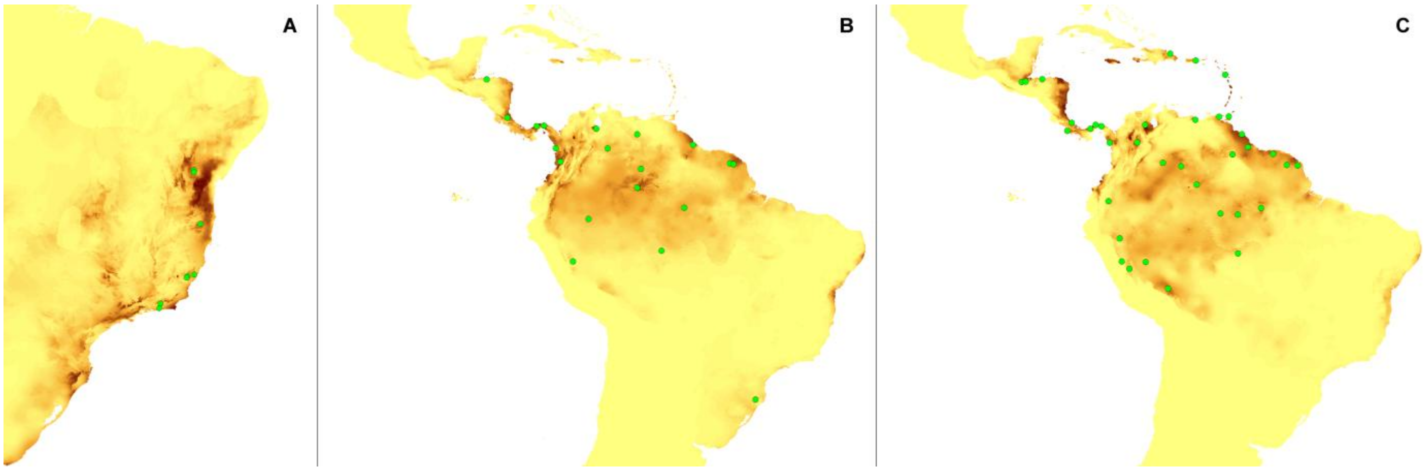

Supplementary Figure S2. Current distribution of suitable climatic niches of South American orchids included in the study: *Pogoniopsis schenckii* (**A**), *Uleiorchis ulaei* (**B**), *Wullschlaegelia calcarata* (**C**). Maps were generated in ArcGis 9.3[76] (http://www.esri.com/).

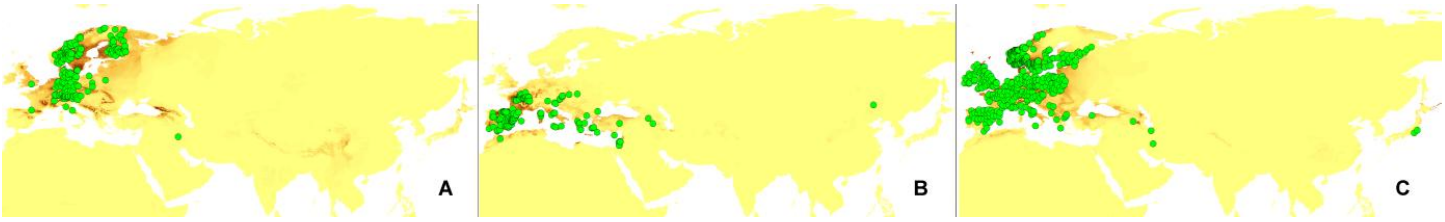

Supplementary Figure S3. Current distribution of suitable climatic niches of European orchids included in the study: *Neottia nidus-avis* (**A**), *Epipogium aphyllum* (**B**), *Limodorum abortivum* (**C**). Maps were generated in ArcGis 9.3[76] (http://www.esri.com/).

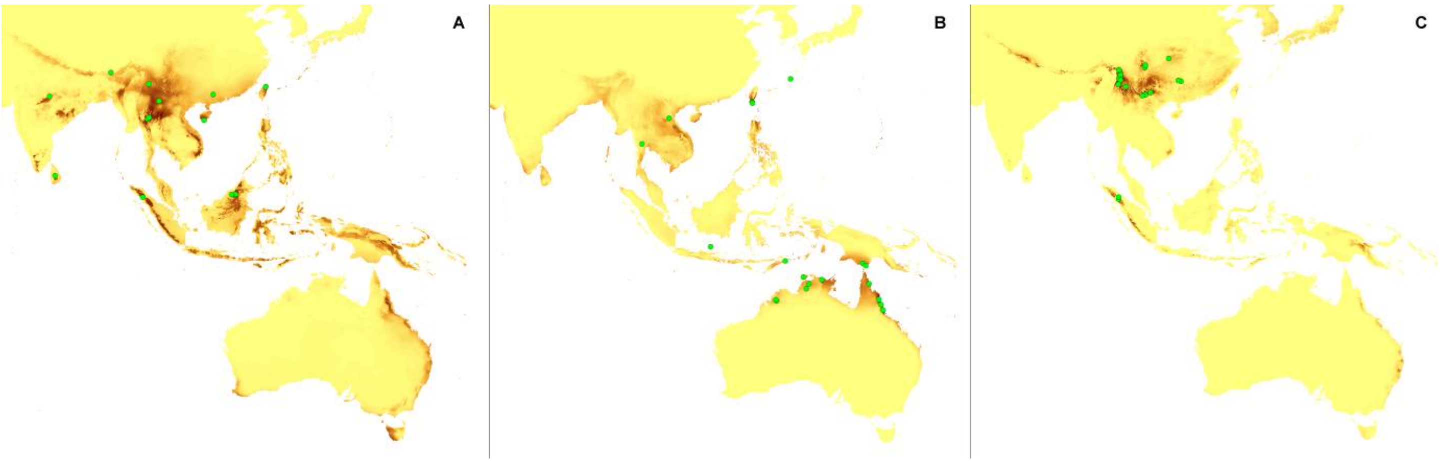

Supplementary Figure S4. Current distribution of suitable climatic niches of Asian orchids included in the study: *Aphyllorchis montana* (**A**), *Didymoplexis pallens* (**B**), *Galeola lindleyana* (**C**). Maps were generated in ArcGis 9.3[76] (http://www.esri.com/).

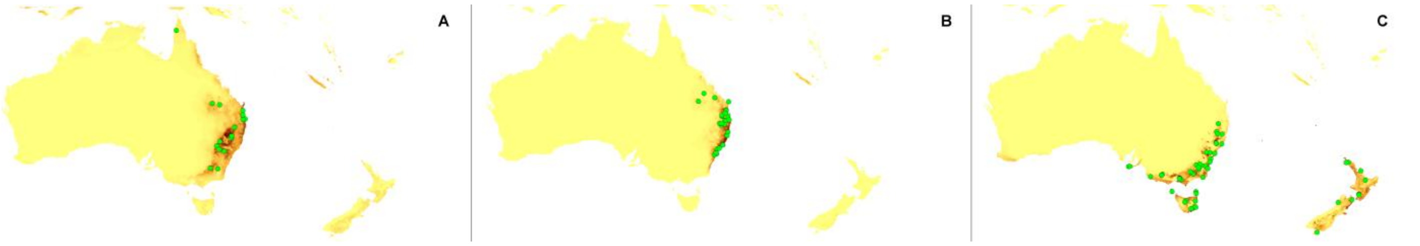

Supplementary Figure S5. Current distribution of suitable climatic niches of Australian orchids included in the study: *Dipodium hamiltonianum* (**A**), *Erythrorchis cassythoides* (**B**), *Gastrodia sesamoides* (**C**). Maps were generated in ArcGis 9.3[76] (http://www.esri.com/).

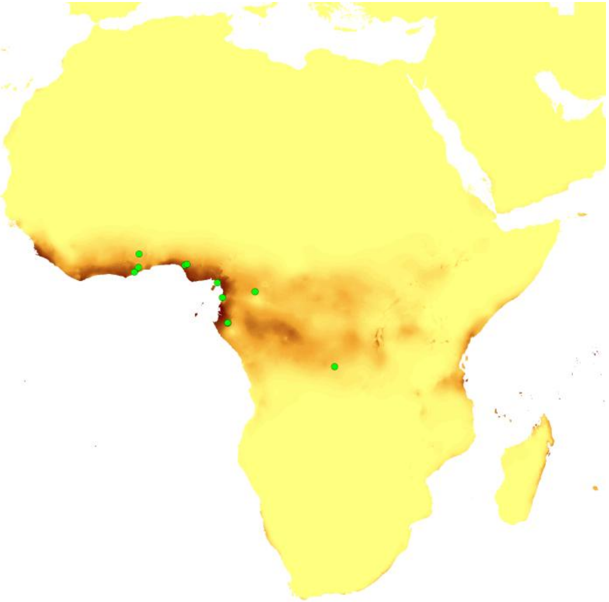

Supplementary Figure S6. Current distribution of suitable climatic niches of African *Auxopus macranthus*. Maps were generated in ArcGis 9.3[76] (http://www.esri.com/).

Supplementary Table S1. The average training AUC and the standard deviation (SD) values for ENM analyses.

Region	Species	Present		A1b		A2a		B2a	
		**AUC**	**SD**	**AUC**	**SD**	**AUC**	**SD**	**AUC**	**SD**
North America	*Cephalanthera austiniae*	0.992	0.001	0.992	0.001	0.992	0.001	0.992	0.001
	Corallorhiza wisteriana	0.979	0.002	0.975	0.003	0.975	0.003	0.978	0.003
	Hexalectris spicata	0.980	0.004	0.980	0.004	0.980	0.004	0.979	0.005
South America	*Pogoniopsis schenckii*	0.978	0.005	0.978	0.005	0.979	0.005	0.978	0.005
	Uleiorchis ulaei	0.948	0.017	0.944	0.017	0.946	0.017	0.946	0.017
	Wullschlaegelia calcarata	0.953	0.009	0.950	0.009	0.950	0.009	0.952	0.009
Australia	*Dipodium hamiltonianum*	0.986	0.005	0.987	0.005	0.987	0.005	0.987	0.005
	Erythrorchis cassythoides	0.994	0.001	0.993	0.001	0.994	0.001	0.993	0.001
	Gastrodia sesamoides	0.981	0.003	0.982	0.002	0.982	0.003	0.981	0.003
Asia	*Aphyllorchis montana*	0.969	0.011	0.969	0.011	0.968	0.012	0.967	0.011
	Didymoplexis pallens	0.977	0.008	0.976	0.008	0.976	0.008	0.977	0.008
	Galeola lindleyana	0.989	0.003	0.990	0.002	0.989	0.003	0.989	0.003
Europe	*Neottia nidus-avis*	0.962	0.001	0.961	0.001	0.960	0.001	0.961	0.001
	Epipogium aphyllum	0.973	0.001	0.973	0.002	0.972	0.002	0.973	0.001
	Limodorum abortivum	0.987	0.001	0.986	0.001	0.986	0.001	0.986	0.001
Africa	*Auxopus macranthus*	0.980	0.008	0.979	0.008	0.979	0.007	0.977	0.008

Supplementary Table S2. Estimates of relative contributions of the environmental variables to the Maxent models.

Species	Present			A1b			A2a			B2a		
	**var 1**	**var 2**	**var 3**	**var 1**	**var 2**	**var 3**	**var 1**	**var 2**	**var 3**	**var 1**	**var 2**	**var 3**
Cephalanthera austiniae	bio19 - 35.3	bio18 - 27.7	bio8 - 15.9	bio19 - 37.5	bio18 - 29.5	bio8 - 12.5	bio19 - 37.6	bio18 - 28.8	bio8 - 13.5	bio19 - 37.5	bio18 - 30	bio8 - 13.5
Corallorhiza wisteriana	bio5 - 19.2	bio4 - 18.6	bio2 - 15.8	bio5 - 18.1	bio4 - 17.8	bio2 - 14.9	bio4 - 19	bio5 - 17.8	bio2 - 16	bio4 - 18.8	bio5 - 17.8	bio2 - 15.3
Hexalectris spicata	bio14 - 39	bio1 - 31.2	bio5 - 9.3	bio14 - 39.5	bio1 - 34.1	bio5 - 7.8	bio14 - 38.8	bio1 - 38.2	bio4 - 4.5	bio14 - 39.1	bio1 - 31.2	bio5 - 10.3
Pogoniopsis schenckii	bio5 - 43.7	bio2 - 19.1	bio19 - 18.5	bio5 - 42.5	bio2 - 19.4	bio19 - 18.2	bio5 - 44.3	bio2 - 19.4	bio19 - 18.3	bio5 - 42.9	bio2 - 19.2	bio19 - 18
Uleiorchis ulaei	bio19 - 43.6	bio14 - 9.9	bio2 - 9.6	bio19 - 43.1	bio14 - 11.2	bio2 - 10.4	bio19 - 44.3	bio14 - 11.1	bio2 - 10.2	bio19 - 44.8	bio2 - 11.1	bio14 - 10.1
Wullschlaegelia calcarata	bio14 - 24.2	bio19 - 16.9	bio12 - 15.1	bio14 - 23.2	bio19 - 17.1	bio12 - 16.3	bio14 - 24	bio19 - 16.9	bio12 - 16.6	bio14 - 21.7	bio19 - 19.7	bio12 - 15.4
Dipodium hamiltonianum	bio14 - 41.7	bio12 - 25	bio4 - 8.7	bio14 - 42	bio12 - 24.7	bio4 - 9.3	bio14 - 39.9	bio12 - 25.1	bio4 - 9.3	bio14 - 39.8	bio12 - 25.8	bio4 - 9.9
Erythrorchis cassythoides	bio14 - 32.3	bio18 - 29.9	bio1 - 13.8	bio14 - 33.7	bio18 - 30.2	bio1 - 11.9	bio14 - 33.7	bio18 - 29.6	bio1 - 12.9	bio14 - 33.8	bio18 - 30.1	bio1 - 12.5
Gastrodia sesamoides	bio19 - 37.3	bio14 - 21.6	bio5 - 19.9	bio19 - 40.6	bio14 - 21.7	bio5 - 18.3	bio19 - 35.8	bio14 - 24.2	bio5 - 19.7	bio19 - 37	bio14 - 21.5	bio5 - 20.
Aphyllorchis montana	bio12 - 29.4	bio14 - 16.6	bio4 - 12.9	bio12 - 30.6	bio14 - 15.7	bio4 - 12.3	bio12 - 29.2	bio14 - 16.2	bio4 - 11.4	bio12 - 29.5	bio14 - 17.1	bio1 - 11.7
Didymoplexis pallens	bio4 - 22.3	bio12 - 15.2	bio13 - 15	bio4 - 23.2	bio13 - 14.9	bio12 - 14	bio4 - 21.4	bio13 - 15.5	bio12 - 14.7	bio4 - 24.4	bio13 - 15.3	bio12 - 14.2
Galeola lindleyana	bio18 - 40.9	bio1 - 21.5	bio14 - 9.5	bio18 - 40.2	bio1 - 20.3	bio14 - 10.6	bio18 - 39.8	bio1 - 22.1	bio14 - 10.6	bio18 - 41.9	bio1 – 21.8	bio14 – 9.8

Neottia nidus-avis	bio14 - 32.7	bio4 - 19.3	bio15 - 16.7	bio14 - 32	bio4 - 19.6	bio15 - 15.8	bio14 - 31.8	bio4 - 20.3	bio15 - 17.7	bio14 - 31.3	bio4 - 19.4	bio15 - 16.9
Epipogium aphyllum	bio14 - 37.3	bio5 - 24.2	bio4 - 22	bio14 - 35.9	bio5 - 25.2	bio4 - 22.6	bio14 - 34.6	bio5 - 25.2	bio4 - 23.3	bio14 - 35.5	bio5 - 25.6	bio4 - 22.9
Limodorum abortivum	bio4 - 27.8	bio19 - 27.8	bio1 - 10.5	bio4 - 27.6	bio19 - 27.5	bio5 - 10.9	bio4 - 27.9	bio19 - 25.7	bio5 - 11.6	bio4 - 28.4	bio19 - 26.6	bio1 - 11.3
Auxopus macranthus	bio2 - 35.8	bio4 - 27.2	bio12 - 19.5	bio2 - 38.3	bio4 - 25.5	bio12 - 20.7	bio2 - 35	bio12 - 24.8	bio4 - 24.6	bio2 - 34.1	bio12 - 26	bio4 - 24.2

Supplementary Table S3. Coverage of suitable climatic niches for studied species calculated for the present time and for three scenarios of climate changes.

Region	Species	Coverage of the most suitable (>0.7) habitats [km^2]			
		Present	A1b	A2a	B2a
North America	*Cephalanthera austiniae*	9880.34	0	0	0
	Corallorhiza wisteriana	105959.6	4561.82	2853.84	174019.4
	Hexalectris spicata	104165.2	1355682	1723676	1037501
South America	*Pogoniopsis schenckii*	64622.18	108.1	5426.62	18895.88
	Uleiorchis ulaei	59692.82	2431645	4438997	1332635
	Wullschlaegelia calcarata	110607.9	3168389	2330355	1315209
Australia	*Dipodium hamiltonianum*	32343.52	71021.7	147059.2	151815.6
	Erythrorchis cassythoides	14939.42	129.72	7134.6	1816.08
	Gastrodia sesamoides	11350.5	497.26	7199.46	41877.94
Asia	*Aphyllorchis montana*	103516.6	3999.7	13858.42	24495.46
	Didymoplexis pallens	31975.98	179078.5	129201.1	76815.86
	Galeola lindleyana	85831.4	88577.14	1362.06	7891.3
Europe	*Neottia nidus-avis*	12842.28	519420.5	137676.2	95906.32
	Epipogium aphyllum	211984.1	702239.2	215292	87842.06
	Limodorum abortivum	49596.28	10745.14	23846.86	2486.3
Africa	*Auxopus macranthus*	188656.1	363734.9	584604.8	563655

Supplementary Table S4. Climatic variables used in the analyses.

Code	Description
bio1	Annual Mean Temperature
bio2	Mean Diurnal Range = Mean of monthly (max temp − min temp)
bio3	Isothermality (bio2/bio7) * 100
bio4	Temperature Seasonality (standard deviation * 100)
bio5	Max Temperature of Warmest Month
bio8	Mean Temperature of Wettest Quarter
bio12	Annual Precipitation
bio13	Precipitation of Wettest Month
bio14	Precipitation of Driest Month
bio15	Precipitation Seasonality (Coefficient of Variation)
bio18	Precipitation of Warmest Quarter
bio19	Precipitation of Coldest Quarter

Supplementary Table S5. Background extension in ENM analyses.

Region	Latitude	Longitude
North America	10.35-55.42 N	50.51-140.36 W
South America	37.82 S-26.14 N	27.67-107.97 W
Europe	6.79-78.99 N	11.33 W-148.6 E
Asia	46.63 S-41.01 N	69.73-160.2 E
Australia	8.79-49.09 S	110.66-179.82 E
Africa	39.42 S-38.88 N	22.53 W-57.18 E
Pogoniopsis schenckii	36.83 S-0.66 N	32.25-55.37 W

Supplementary Dataset S1. All localities of the studied species gathered during the studies.

Supplementary Dataset S2. Selected localities of studied species with information on the values of variables used in used in the analyses.
